# Advances in the diagnosis and classification of gastric and intestinal motility disorders

**DOI:** 10.1038/nrgastro.2018.7

**Published:** 2018-04-06

**Authors:** Jutta Keller, Gabrio Bassotti, John Clarke, Phil Dinning, Mark Fox, Madhusudan Grover, Per M. Hellström, Meiyun Ke, Peter Layer, Carolina Malagelada, Henry P. Parkman, S. Mark Scott, Jan Tack, Magnus Simren, Hans Törnblom, Michael Camilleri

**Affiliations:** 1 Israelitic Hospital, Academic Hospital University of Hamburg, Orchideenstieg 14, 22297 Hamburg, Germany.; 2 University of Perugia, Piazza dell’Università, 1, 06121 Perugia, Italy.; 3 Stanford University, 900 Blake Wilbur Dr, Palo Alto, CA 94304, USA.; 4 Flinders Medical Centre, GPO Box 2100, Adelaide 5001, Australia.; 5 University Hospital Zürich, Rämistrasse 100, 8091 Zürich, Switzerland, and St. Claraspital, Kleinriehenstrasse 30, 4058 Basel, Switzerland.; 6 Mayo Clinic, 200 First Street SW, Rochester, MN 55902, USA.; 7 Uppsala University Hospital, Building 40, SE‑75185, Uppsala, Sweden.; 8 Peking Union Medical College Hospital, No.1 Shuaifuyuan Wangfujing Dongcheng District, Beijing, 100730, China.; 9 University of Barcelona, Passeig de la Vall d’Hebron, 119–129, 08035 Barcelona, Spain.; 10 Temple University Hospital, 3401 N Broad St, Philadelphia, PA 19140, USA.; 11 Queen Mary University of London, The Wingate Institute, 26 Ashfield Street, Whitechapel, London E1 2AJ, UK.; 12 University Hospital Gasthuisberg, University of Leuven, Herestraat 49, 3000 Leuven, Belgium.; 13 Sahlgrenska Academy, University of Gothenburg, Blå stråket 5, 41345 Gothenburg, Sweden.

## Abstract

Disturbances of gastric, intestinal and colonic motor and sensory functions affect a large proportion of the population worldwide, impair quality of life and cause considerable health-care costs. Assessment of gastrointestinal motility in these patients can serve to establish diagnosis and to guide therapy. Major advances in diagnostic techniques during the past 5–10 years have led to this update about indications for and selection and performance of currently available tests. As symptoms have poor concordance with gastrointestinal motor dysfunction, clinical motility testing is indicated in patients in whom there is no evidence of causative mucosal or structural diseases such as inflammatory or malignant disease. Transit tests using radiopaque markers, scintigraphy, breath tests and wireless motility capsules are noninvasive. Other tests of gastrointestinal contractility or sensation usually require intubation, typically represent second-line investigations limited to patients with severe symptoms and are performed at only specialized centres. This Consensus Statement details recommended tests as well as useful clinical alternatives for investigation of gastric, small bowel and colonic motility. The article provides recommendations on how to classify gastrointestinal motor disorders on the basis of test results and describes how test results guide treatment decisions.

Disturbances of gastric and intestinal motor functions such as gastroparesis, functional dyspepsia, enteric dysmotility, IBS and constipation affect a large proportion of the population worldwide, impair quality of life and cause considerable health-care costs^1,2^. Assessment of gastrointestinal motility in these patients can serve to establish diagnosis and to guide therapy. Comprehensive consensus papers published in 2008 (REF. 3) and 2011 (REF. 4) detailed how to evaluate and interpret gastric, small intestinal and colonic motility by intraluminal measurements and transit tests in clinical practice.

Advances in diagnostic techniques for the evaluation of gastrointestinal motor function necessitate an update about indications for and selection and performance of currently available tests, how motility disorders can be differentiated and classified based on these tests and how the results guide treatment decisions, as noted in this Consensus Statement. A panel of international motility experts has re-examined these issues and provides concise information on test principles, practical performance and interpretation of individual tests (BOX 1). Further details on these topics will be provided in technical position statements that will be published separately.

## Methods

This Consensus Statement is part of a series of papers on gastrointestinal motility initiated by the International Working Group for Disorders of Gastrointestinal Motility and Function. Authors were invited based on their experience and reputation in the field and chosen to cover the intended scope of the manuscript; they represent experts from many European countries, North America, Australia and China. Experts on gastric, small bowel and colonic motility disorders first developed statements regarding evaluation of transit and contractility of the respective segments of the gastro intestinal tract, which were based on already available consensus statements^3,4^. Consensus statements from other gastrointestinal societies or expert groups were searched for and included when appropriate, for example, if they were published more recently or if they covered relevant areas not specifically addressed in the previous documents^3,4^. Moreover, an updated literature search using PubMed and Medline was performed centrally by the corresponding author (J.K.) with additions from the co-authors. The literature search started on the date specified as the end date of the liteature searches performed for two previously published consensus papers^3,4^ (that is, 1 Jan 2008 for intraluminal measurements of gastrointestinal motility and 1 Jan 2010 for transit tests); the search covered the period until 14 Apr 2016 and was generally limited to human studies. The literature search revealed 1,111 publications, of which 202 were selected on the basis of study quality (which did not include a formal evaluation, but the level of evidence was assessed in line with the Oxford Centre for Evidence-based Medicine (https://www.cebm.net/2009/06/oxford-centre-evidence-based-medicine-levels-evidence-march-2009/) and were made available to all authors. Low-quality studies were also considered if the topic was deemed relevant and not covered otherwise. The literature search on gastric emptying included the following terms, revealing 624 papers: “gastric emptying”, “gastro paresis”, “dumping”, “measurement”, “test”, “evaluation” and “diagnosis”. The literature search on intra luminal tests of gastric motility included the following terms, revealing 67 papers: “gastric”, “antral”, “antroduodenal”, “antroduodenojejunal”, “motility”, “contraction”, “intraluminal” and “manometry”. The literature search on small bowel transit included the following terms, revealing 130 papers: “orocaecal transit”, “OCCT”, “small bowel transit”, “intestinal transit”, “measurement”, “test”, “evaluation”, “diagnosis”, “sensitivity” and “specificity”. The literature search on intraluminal tests of small bowel motility included the following terms, revealing 13 papers: “small bowel”, “antroduodenal”, “antroduodenojejunal”, “intestinal”, “intraluminal”, “motility” and “manometry”.

The literature search on colonic transit included the following terms, revealing 222 papers: “colonic transit”, “Hinton”, “measurement”, “test”, “evaluation”, “diagnosis”, “sensitivity” and specificity. The literature search on intraluminal tests of colonic motility included the following terms, revealing 55 papers: “colonic”, “motility”, “contraction”, “intraluminal” and “manometry”.

Statements were distributed via e-mail, and each author had to confirm full agreement, minor concerns or disagreement in writing. Concerns or disagreement had to be explained. All statements with at least one author in disagreement or more than three authors with minor concerns were modified after discussion in conference calls and in a face-to-face meeting at United European Gastroenterology Week in Vienna, Austria, in October 2016. It was required that no more than one author disagree with the final Consensus Statement for it to be included in the final version of the manuscript. Statements contain crucial information on the respective topic and/or give recommendations on when or how to perform and interpret motility tests. They are marked as bold bulleted points throughout the manuscript. All authors consented to the final version of the manuscript, including comments.

## Clinical application of motility testing

• Before investigation of gastrointestinal motor function, mucosal or structural diseases such as inflammatory or malignant disease should be excluded.

Symptoms of gastrointestinal motor disorders are nonspecific: dysmotility cannot be differentiated from inflammatory or malignant disease on the basis of patient history alone. For example, epigastric pain, early satiety and abdominal fullness are typical symptoms of gastro paresis but can also be due to gastroduodenal ulcers or gastric cancer. Moreover, inflammatory diseases of the small and large bowel are associated with delayed gastric emptying, which can be reversible after treatment of inflammation^5,6^. Thus, it is important to first exclude other aetiologies, in particular, mucosal and obstructive lesions, by appropriate investigations such as upper and lower gastrointestinal endoscopy, imaging techniques and laboratory investigation. Such tests are mandatory in patients with ‘red flags’ (that is, weight loss, low haemoglobin levels and substantial episodes of vomiting) but should also be performed if the motility tests are invasive or symptoms are severe. In patients with moderate complaints and no alarm symptoms, noninvasive motility testing might be considered. The selection of tests will also be influenced by availability and the costs of diagnostic procedures in different health-care systems.

In general, motility investigations are usually limited to patients with relevant complaints that can be related to dysmotility and that markedly affect quality of life, nutrition, social function or work productivity and, rarely, to increased mortality^7,8^. As with any diagnostic procedure, they are justified only if the results can be expected to influence clinical management.

## Investigation of gastric motor function

### Indications and clinical importance

Tests of gastric motor function comprise gastric emptying tests and intraluminal measurements of contractility.

• Clinical investigation of gastric motor function is indicated in patients in whom upper gastrointestinal endoscopy is normal or does not provide a definitive diagnosis and in patients in whom there is suspicion of gastroparesis, unexplained nausea and vomiting or dumping syndrome.

• Abdominal symptoms of accelerated and delayed gastric emptying are similar, such that gastric emptying tests can be necessary for delineation of motor dysfunction.

Symptoms suggestive of delayed gastric emptying include early satiety, nausea, vomiting, regurgitation, bloating, postprandial fullness, visible upper abdominal distention, abdominal pain and weight loss^1,9^. Most patients with rapid gastric emptying present with abdominal symptoms that mimic those of gastro pare-sis^10,11^. Suspicion of gastroparesis is further supported by identifying risk factors, for example, long-standing diabetes mellitus^9^. Conversely, suspicion of gastric dumping is supported by a history of upper gastrointestinal surgery^12^. However, in a large retrospective study in >600 patients with dyspepsia, the majority of patients with symptoms in association with rapid gastric emptying had no identifiable cause^11^. Furthermore, upper gastrointestinal symptoms had a poor clinical specificity relative to the actual rate of gastric emptying on scintigraphy, underlining the need for function testing to guide treatment. In particular, the positive predictive value of clinical suspicion for delayed gastric emptying was only 29%^11^.

• The diagnosis of gastroparesis requires objective evidence of clearly delayed gastric emptying in symptomatic patients.

**Figure 1 |:**
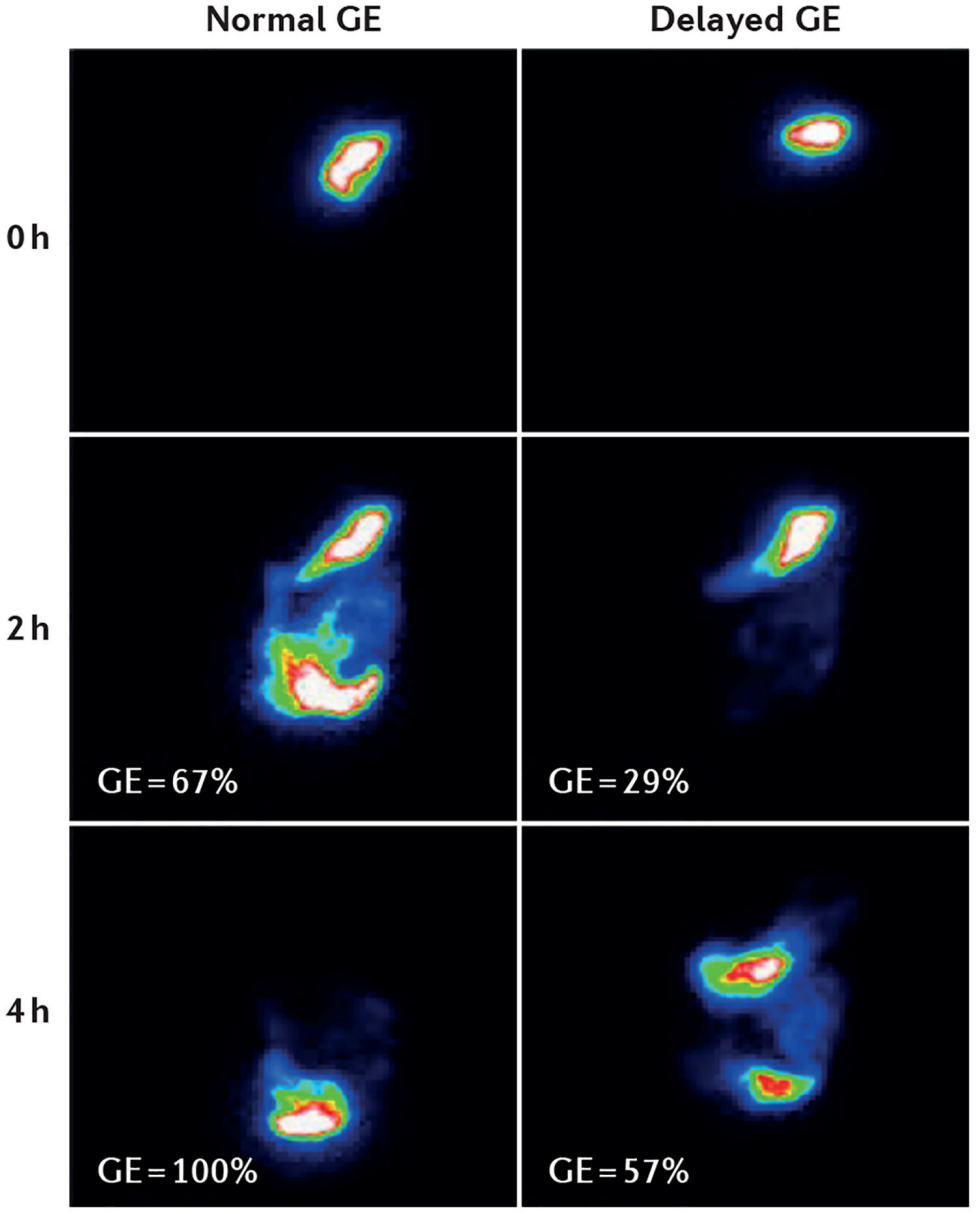
Representative examples of gastric emptying as assessed using scintigraphy. Standardized scintigraphic study of gastric emptying of solids with consumption of a 320 kcal radiolabelled meal (scrambled eggs labelled with ^99m^Tc; Mayo Clinic protocol^30^) and imaging over 4 h. In the individual with normal gastric emptying (GE) (left panel), large amounts of the meal are emptied from the stomach at 2 h, and GE is completed at4 h. In the individual with delayed GE (right panel), gastric retention of the test meal at 2 h and particularly at 4 h is increased (normative values were determined from 319 healthy volunteers; clinically relevant delayed GE is defined as a percentage retention >75% at 2 h and >25% at 4 h)^30^.

Because accelerated, normal and delayed gastric emptying cannot be differentiated reliably based on type or severity of gastrointestinal symptoms, objective measurement of clearly delayed gastric emptying (gastric emptying time increased above the upper level of normal) using well-validated techniques such as gastric emptying scintigraphy (FIG. 1) is required for diagnosis of gastroparesis. To obtain a more specific symptom pattern and a better separation from functional dyspepsia with delayed emptying, gastroparesis has been proposed to require a stricter definition (for example, >3 standard deviations above the mean value in healthy volunteers)^13^.

The merit of gastric emptying studies for clinical management has been questioned because of variations in the reports of association between gastric emptying rates and symptoms. Several studies published during the past 7 years have shown a positive association between symptoms of gastroparesis and gastric emptying times^14–19^. Measurement of gastric emptying can also predict responsiveness to different therapeutic options^20,21^. For example, the presence of slow gastric emptying in patients with functional dyspepsia was associated with poor response to antidepressant medications that target visceral hypersensitivity^21^. On the other hand, one systematic literature review that used multiple methods, various symptom instruments and diverse treatments showed that most drugs that improved idiopathic and diabetic gastroparesis failed to show a statistically significant relationship with the degree of symptom improvement and acceleration of gastric emptying across studies^22^.

Some groups have observed that the association between clinical improvement and acceleration of gastric emptying depends on the aetiology of gastroparesis^20,23^, which could partly explain inconsistent findings across studies^22^. Moreover, the modes of action of drugs used for acceleration of gastric emptying are extremely heterogeneous and potentially induce dysfunctions that cause symptoms. For example, motilin receptor agonists markedly accelerate gastric emptying but simultaneously impair gastric accommodation and can induce dyspeptic symptoms^1^. Several additional factors other than a global delay in gastric emptying — such as antral distension, antral hypomotility, gastric dysrhythmias, visceral hypersensitivity or psychological disturbances — could explain, in part, the symptoms experienced by patients with gastroparesis^24^.

• In patients with upper gastrointestinal surgery, diagnosis of dumping syndrome can be made on the basis of typical symptoms and findings such as post prandial hypoglycaemia or hypotension. In unclear cases, provocation tests that prove dumping syndrome are the basis of diagnosis, which is supported by evidence of accelerated gastric emptying, preferably of liquids.

**Figure 2 |:**
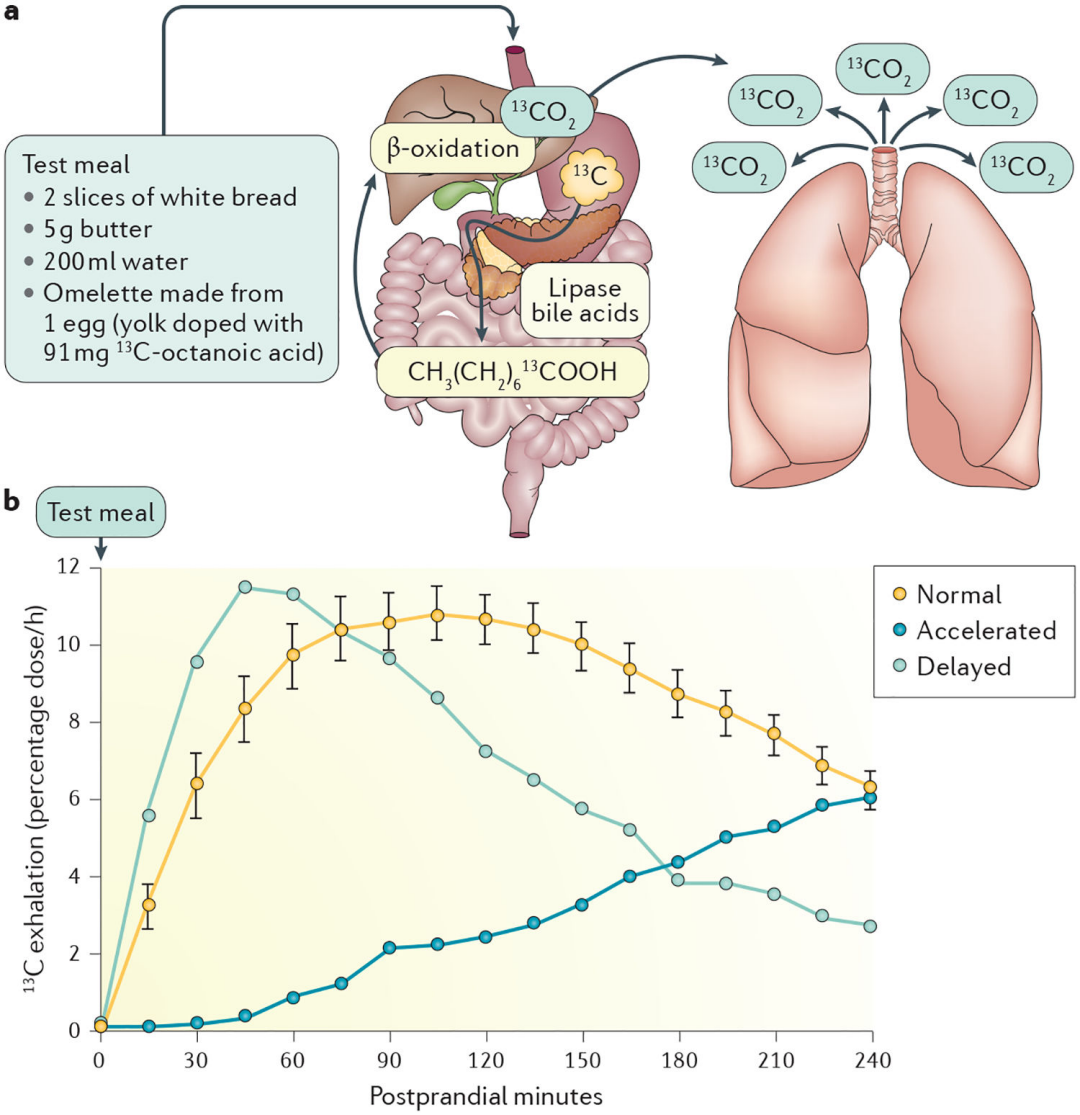
^13^C-octanoic acid gastric emptying breath test. The test principle underlying the ^13^C-octanoic acid breath test (part **a**) is as follows: ^13^C-octaonoic acid is rapidly absorbed after gastric emptying and transported to the liver. Hepatic metabolism leads to production and exhalation of ^13^CO_2_. Thus, alterations of the ^13^C:^12^C ratio in breath samples collected at multiple time points postprandially reflect gastric emptying. Examples (part **b**) of values for accelerated, normal and delayed gastric emptying are shown. Normal data (mean ± s.e.m.) are derived from 20 healthy individuals^6^.

Dumping syndrome is a common complication of oesophageal, gastric or bariatric surgery and includes early and late dumping symptoms^12^. Early dumping occurs within 1 h after eating, when rapid emptying of food into the small intestine triggers rapid fluid shifts into the intestinal lumen and release of gastrointestinal hormones, resulting in gastrointestinal and vasomotor symptoms. Late dumping occurs 1–3 h after carbohydrate ingestion and is caused by an incretin-driven hyperinsulinaemia. According to clinical experience, in patients with typical symptoms after surgery, gastric emptying tests (FIGS 1,2) usually add little to the diagnosis. However, nearly 80% of patients with rapid gastric emptying according to scintigraphy had no identifiable underlying cause for the accelerated emptying even though one-quarter of these patients had associated hypoglycaemia^11^. Liquid test meals might better detect acceleration of early gastric emptying; studies using solid meals generally have low sensitivity and specificity for detecting accelerated gastric emptying^12,25^.

• Investigation of gastric emptying can be useful in the following situations: poorly controlled diabetes mellitus; severe GERD unresponsive to acid suppressants (particularly before fundoplication); systemic sclerosis; after lung transplantation; Parkinson disease; generalized gastrointestinal motility disorders; and patients under consideration for intestinal or colonic surgery or transplantation because of motility disorders.

In these conditions, delayed gastric emptying can be clinically relevant even without typical symptoms of gastro paresis, as the test identifies gastric dysfunction that could have clinical or therapeutic implications. Thus, impaired coordination between nutrient delivery to the duodenum and onset of insulin effect can impair glycaemic control in patients with insulin-dependent diabetes and gastroparesis^9^. Delayed gastric emptying could cause gastro-oesophageal reflux and regurgitation in a subset of patients with GERD^26^ and systemic sclerosis^27^. Lung transplant recipients can have markedly impaired gastric emptying (secondary to vagal injury) with a risk of aspiration and post-transplant sequelae^28^. Delayed gastric emptying contributes substantially to fluctuations in symptom control in patients with Parkinsonism on long-term levodopa therapy^29^. In patients with generalized gastrointestinal motility disorders, particularly in those under consideration for abdominal surgery because of the motility dis order (for example, colonic inertia), knowledge of gastric involvement is required to individualize therapy.

• Investigation of antral or antropyloroduodenal contraction patterns should be considered in patients with severely impaired function and marked symptoms in whom knowledge of the pathophysiology and/or severity of a gastric or gastrointestinal motility disorder is required for patient management.

Detailed investigation of gastric contractility generally requires invasive techniques such as intraluminal manometry (including stomach and small bowel) and should, therefore, be limited to patients with severe symptoms. Clinically relevant information includes identification of gastric involvement in systemic sclerosis with reduced antral contraction amplitude (on average, <40 mmHg) and the selection of dietary recommendations and identification of sites for enteral feeding^3^ (for example, in the jejunum in patients with severe antral hypomotility).

### Recommended diagnostic approaches

• Scintigraphy is the reference standard for measurement of gastric emptying.

A consensus report^24^ has recommended a standardized protocol for the performance of gastric emptying scintigraphy in the USA and has provided normal values. Accordingly, gastric emptying scintigraphy should be performed with a low-fat, egg white meal (~240 kcal, 2% fat) with imaging at 0 h, 1 h, 2 h and 4 h to assess emptying of solids. The 1 h scan is used to detect rapid gastric emptying (percentage retention <30%) and the 2 h and 4 h scans are used to detect delayed gastric emptying (retention >60% or >10%, respectively). A second well-validated protocol has been established by the Mayo Clinic, USA^30^, and uses a 320 kcal, 30% fat meal (FIG. 1). However, even in the USA, despite society guidelines, many centres continue to perform suboptimal studies (duration 1–2 h) that undermine the quality and utility of the test^4^. In most other countries, including the European ones, there are no widely accepted standard procedures. For interpretation of test results, it has to be taken into account that clinical utility depends on complete consumption of adequate test meals and adequate duration of imaging.

• ^13^C-gastric emptying breath tests (^13^C-GEBTs) can be used as an alternative to scintigraphy.

Test meals labelled with the stable, nonradioactive isotope ^13^C can be used to measure gastric emptying. The edible blue–green algae, ^13^C-labelled *Spirulina platensis*^31^ or the medium-chain fatty acid, ^13^C-octanoic acid (^13^C-OA)^32^, is typically used to label solids; ^13^C-acetate is used for liquids^33^. On delivery to the duodenum, the ^13^C-containing substrate is either absorbed directly (^13^C-OA or ^13^C-acetate) (FIG. 2) or digested and then absorbed (^13^C-labelled *S. platensis*). Subsequently, it is metabolized, usually in the liver, and finally excreted by the lungs as ^13^CO_2_. Consequently, ^13^C-GEBTs are indirect tests that involve multiple steps. For ^13^C-acetate, an interaction has been demonstrated between the rate of ^13^C delivery to the duodenum and ^13^C recovery in breath^34^. Moreover, it has been hypothesized that ^13^C-GEBTs might be inaccurate in conditions associated with substantial malabsorption or liver or lung diseases. However, clinical studies do not substantiate this assumption^35^; even in patients with liver cirrhosis (~50% Child–Pugh score C), ^13^C-OA metabolism was found to be normal^36^ and the ^13^C-OA breath test correlated well with scintigraphy in patients who were critically ill^37^.

Intraindividual and interindividual variabilities of all ^13^C-GEBTs are high, but they are similar to the variations observed with scintigraphy^4,31,38^ and, therefore, reflect day-to-day physiological variability in gastric emptying. Results of the ^13^C-labelled *S. platensis* GEBT show a high concordance (*r* = 0.86) with scintigraphic data^31^, and the test was approved by the FDA for the evaluation of gastric emptying in April 2015. The test kit is commercially available (USA only), and the protocol is exactly defined and has been validated in a large group of healthy volunteers and patients^31^. For the ^13^C-OA GEBT and the ^13^C-acetate GEBT, several test protocols and multiple mathematical analysis methods have been proposed^4,32,33,39,40^. When using these tests, it is important to strictly follow a standardized, validated approach.

• Markedly prolonged retention of the wireless motility capsule (WMC) might be a marker of delayed gastric emptying.

**Figure 3 |:**
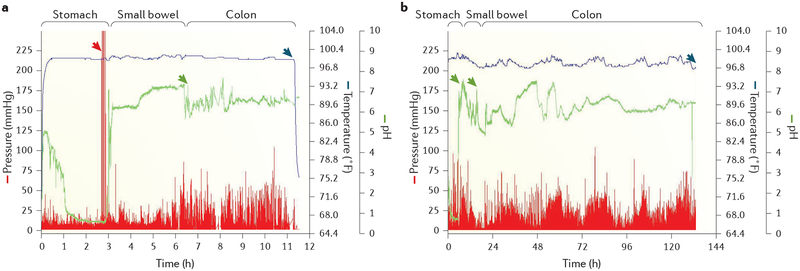
Example wireless motility recording. Wireless motility recordings in a healthy male participant (part **a**) and a female patient with severe constipation (part **b**) are shown. Gastric emptying in the control individual (part **a**) occurs after ~3 h (upper limit of normal: 5–6 h) and is preceded by strong antral contractions suggestive of antral phase III motility (red arrow). A constant decrease in pH at ~6 h 30 min (green arrow) marks ileocaecal transit, such that small bowel transit time is estimated to be ~3 h 30 min (normal range: 2.5–8 h). Abrupt temperature drop (blue arrow) shows that the capsule is excreted after ~11h 30 min, such that colonic transit time is ~5 h, which is equivalent to the lower limit of normal. In the patient with severe constipation (part **b**), gastric emptying time is relatively long (~5 h, first green arrow), ileocaecal transit occurs ~16 h after ingestion of the motility capsule (second green arrow), and excretion of the capsule does not occur until 133 h (blue arrow), such that both small bowel transit time (~11 h) and colonic transit time (~117 h) are prolonged. Please note that the timescales are different for the left and right panels.

**Figure 4 |:**
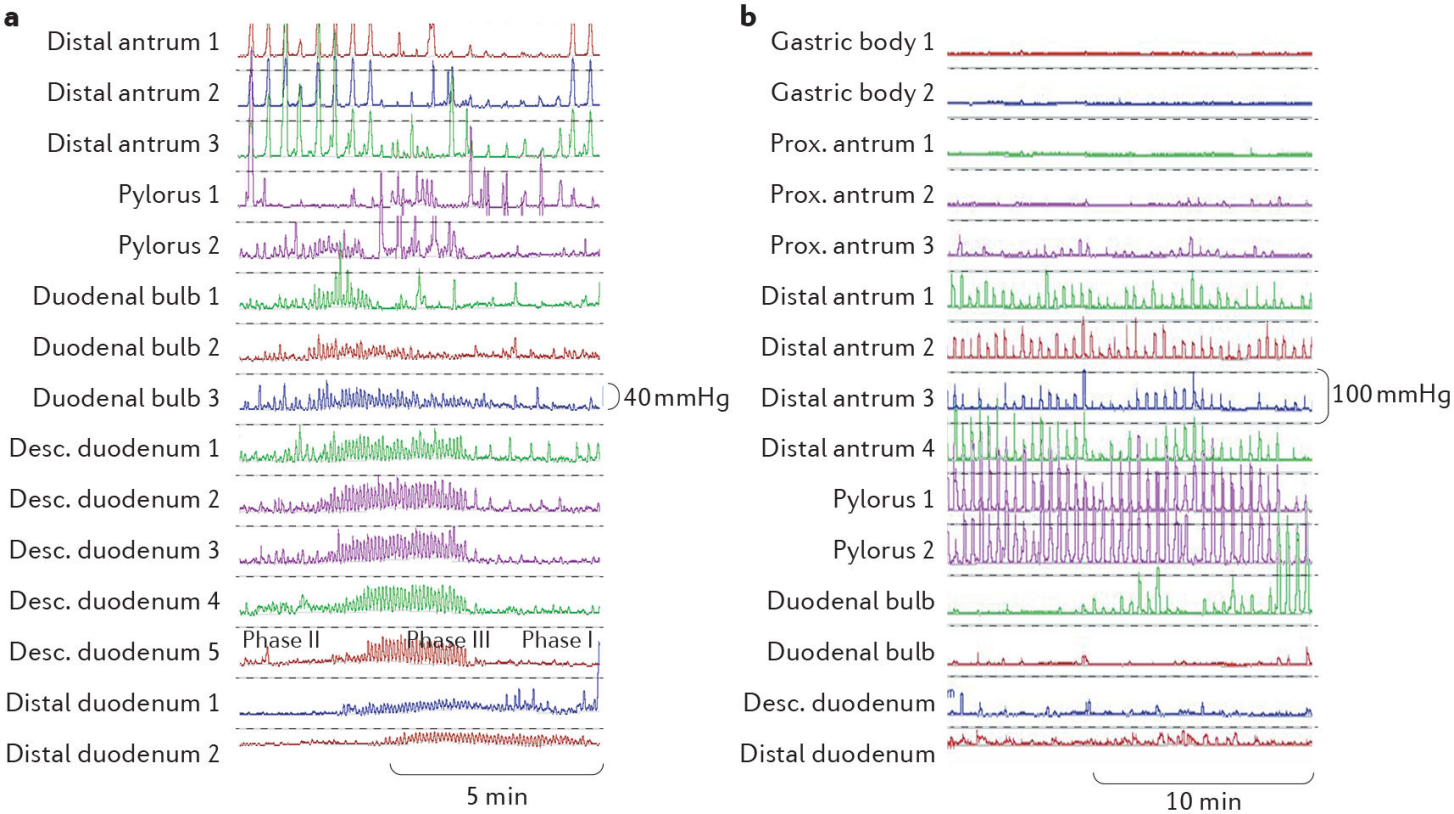
Example plots of high-resolution gastroduodenal manometry. High-resolution gastroduodenal manometry plots are shown for normal fasting (part **a**) and postprandial (part **b**) motility. Antral motility is characterized by high-amplitude contractions with a maximal contraction rate of ~3 per min. Amplitudes of contraction in the small bowel are lower, but frequency is higher (up to ~12 per min). During the fasting state (part **a**), there is a constant transition between phases I to III of the interdigestive migrating motor complex (MMC) with motor quiescence during phase I, irregular contractions that are propagated over only smaller segments during phase II and regular, aborally propagated contractions that usually start in the stomach and traverse long segments of the small bowel during phase III. Postprandially (part **b**), MMC activity is interrupted and replaced by irregular contractions that serve to mix the luminal contents and to slowly propel them towards the more distal intestine. Desc., descending; Prox., proximal.

The WMC (for example, SmartPill, Medtronic, USA) is a single-use, orally ingested, non-digestible, data-recording capsule that measures pH, pressure and temperature throughout the gastrointestinal tract^4^. A marked increase in pH units is used to estimate gastric emptying time (FIG. 3). The WMC has been approved for gastric emptying measurements by the FDA and has a CE mark for the European Union (complies with the European Union safety requirements). However, as the WMC is a large, non-digestible, solid object, it does not empty with the meal but rather is most often cleared from the stomach by powerful interdigestive (migrating motor complex (MMC) phase III) (FIG. 4) antral contractions that occur after the meal has been emptied to clear the stomach of indigestible material^41^. Accordingly, passage of the WMC into the duodenum correlates only modestly with gastric emptying of nutrients^41,42^. Emptying of the capsule is also delayed in patients with reduced or weak MMC phase III contractions. These aspects must be taken into consideration for evaluation of the test.

• Antral or antropyloroduodenal manometry is the reference method for evaluation of gastric contraction patterns.

Catheter-based manometry with multiple pressure sensors located in the antrum, pylorus and duodenum is the only clinically available test that enables detailed assessment of coordinated gastric contraction patterns^3^ (FIG. 4).

## Investigation of small bowel motor function

### Indications and clinical importance

• Tests of small intestinal motility are indicated in patients with suspected severe chronic small bowel dysmotility.

Even patients with very severe small bowel dysmotility fulfilling the diagnostic criteria for chronic intestinal pseudo-obstruction (CIPO) have nonspecific symptoms such as pain (80%), vomiting (75%), constipation (40%) and diarrhoea (20%); this lack of specific symptoms has led to misdiagnosis on initial presentation with mechanical bowel obstruction or treatment-refractory constipation in 80% of patients^43^. Severe small bowel dysmotility is usually identified by chronic disabling gastro intestinal symptoms, which are associated with dilatation of some part of the small bowel, with inconclusive results of endoscopic and radiological investigations or surgical exploration. It is frequently associated with impaired nutritional intake.

• Only those results of intestinal transit tests that deviate substantially from normal values are considered diagnostic of abnormality and indicative of either accelerated or delayed small bowel transit.

Small intestinal transit tests are noninvasive, but their clinical utility is limited by high interindividual and intraindividual variability of small bowel transit in healthy individuals (even by as much as >50%)^4^, which leads to a wide normal range. Experts therefore agree that only abnormal results, based on recorded transit times clearly outside normative ranges, should be considered diagnostic.

• Manometric evaluation of small bowel contraction patterns should be limited to patients with chronic severe and otherwise insufficiently explained symptoms or should be used when knowledge of small bowel motility disturbances is required for management.

Detailed clinical evaluation of antroduodenojejunal contraction patterns by manometry is available in only highly specialized centres (FIG. 4). A WMC can also measure amplitude of antral, small bowel and colonic contractions during its passage through the gastro intestinal tract (FIG. 3). Individual antral contractions detected by the WMC correlated closely with those observed on manometry, and in theory, many of the indications for antroduodenojejunal manometry should also apply to the WMC^3^. However, the WMC records pressure at a single recording site and cannot appraise propagation of contractions. Thus, it is uncertain whether the WMC can be a substitute for catheter-based manometric investigation of small bowel motility in terms of propagation of pressure waves.

• Antroduodenojejunal manometry can serve to exclude major motility disturbances in patients with otherwise equivocal findings.

An entirely normal result in a manometric study suggests that motor dysfunction of the upper gastrointestinal tract is not a cause of patient symptoms^44^ and that it can differentiate a true motility disorder from a somatoform disorder in children^45^.

• Altered small bowel motility on manometry could suggest underlying myopathy or neuropathy. Severe motor pattern alterations in combination with documented episodes mimicking mechanical obstruction enable the diagnosis of CIPO.

Myopathic disorders (for example, systemic sclerosis, amyloidosis and hollow visceral myopathy) are characterized by low-amplitude intestinal contractions (<20 mmHg) at affected intestinal sites^3^. A combination of frequent duodenojejunal MMCs (>3 over 3 h) during the fasting period, absence of antral MMC phase III, presence of postprandial antral hypomotility and a rapid return of MMC activity (within 2 h) after a >400 kcal meal suggests autonomic neuropathy, typically with vagal dysfunction^46^. Other neuropathic disorders have been associated with antral hypomotility, abnormal propagation of MMC phase III, hypercontractility in the duodenojejunum (phase III contraction amplitudes >60–100 mmHg (P.M.H., unpublished data) and failure to generate the fed response^3^. However, studies comparing manometric and histological findings are weak, and in the absence of a gold standard, the sensitivity and specificity of manometry abnormalities for differentiating causes of motility diseases have not been extensively evaluated.

For the identification of manometric patterns that predict obstruction, manometry has been compared with the results of laparotomy^47^. A study published in 1994 confirmed that non-propagated clustered contractions (>30 min duration) and simultaneous prolonged (>8 s) or summated contractions suggest mechanical obstruction even when this finding is equivocal on barium small bowel radiography^47^. However, with modern and more-sensitive imaging techniques such as CT enterography or magnetic resonance enteroclysis, manometry is seldom required for this indication in clinical practice.

CIPO is a rare disease in which severe intestinal dysmotility impairs transit of chyme such that patients present with signs of subileus and ileus on imaging without mechanical obstruction^48,49^. Small intestinal manometry permits diagnosis of severe intestinal motility disturbances compatible with CIPO^50^ even during mostly asymptomatic intervals. Moreover, manometry can be used to determine which organs need to be transplanted (isolated versus multi visceral transplantation) in patients failing all other treatment options^3^.

Additional indications for small bowel manometry include detection of retrograde propagated contractions, for instance, after Roux-en-Y gastric surgery^51^, and exclusion of generalized dysmotility in patients with colonic inertia before subtotal colectomy. This step is relevant because patients with additional upper gastrointestinal motor abnormalities have a worse long-term outcome after surgery^52^. Whereas small bowel manometry can confirm a diagnosis of rumination syndrome^3^, high-resolution oesophageal manometry with impedance^1,53^ is now preferred for this indication.

### Recommended diagnostic approaches

• Scintigraphy is the reference method for evaluation of small bowel transit time.

Scintigraphic assessment of small bowel transit time is usually performed as part of a whole-gut transit study^4^. Scintigraphy directly visualizes passage of the radioactive marker throughout the small bowel and provides physiological and quantitative data. However, the technique is not standardized, has wide normal ranges and is rarely performed outside the USA.

• The WMC can be used to measure small bowel transit.

The WMC uses pH landmarks to identify passage through the pylorus and through the ileocaecal junction for calculation of small bowel transit time (FIG. 3). A small study of ten healthy adults who underwent WMC and scintigraphy simultaneously demonstrated a moderate correlation between the two methods (*r* = 0.69, *P* = 0.05)^54^, but validation studies for the WMC have mostly concentrated on whole-gut transit time, gastric emptying time or colonic transit time. Moreover, in a large study of 215 healthy volunteers published in 2015, the ileocaecal junction could not be clearly identified by WMC based on pH patterns in >10% of healthy individuals, and the agreement between automated software analysis and manual reading was much lower for small bowel transit time than for any other regional or whole-gut transit time^55^.

• The lactulose H_2_ breath test (LHBT) is an inexpensive and noninvasive but less precise alternative marker of small bowel transit.

**Figure 5 |:**
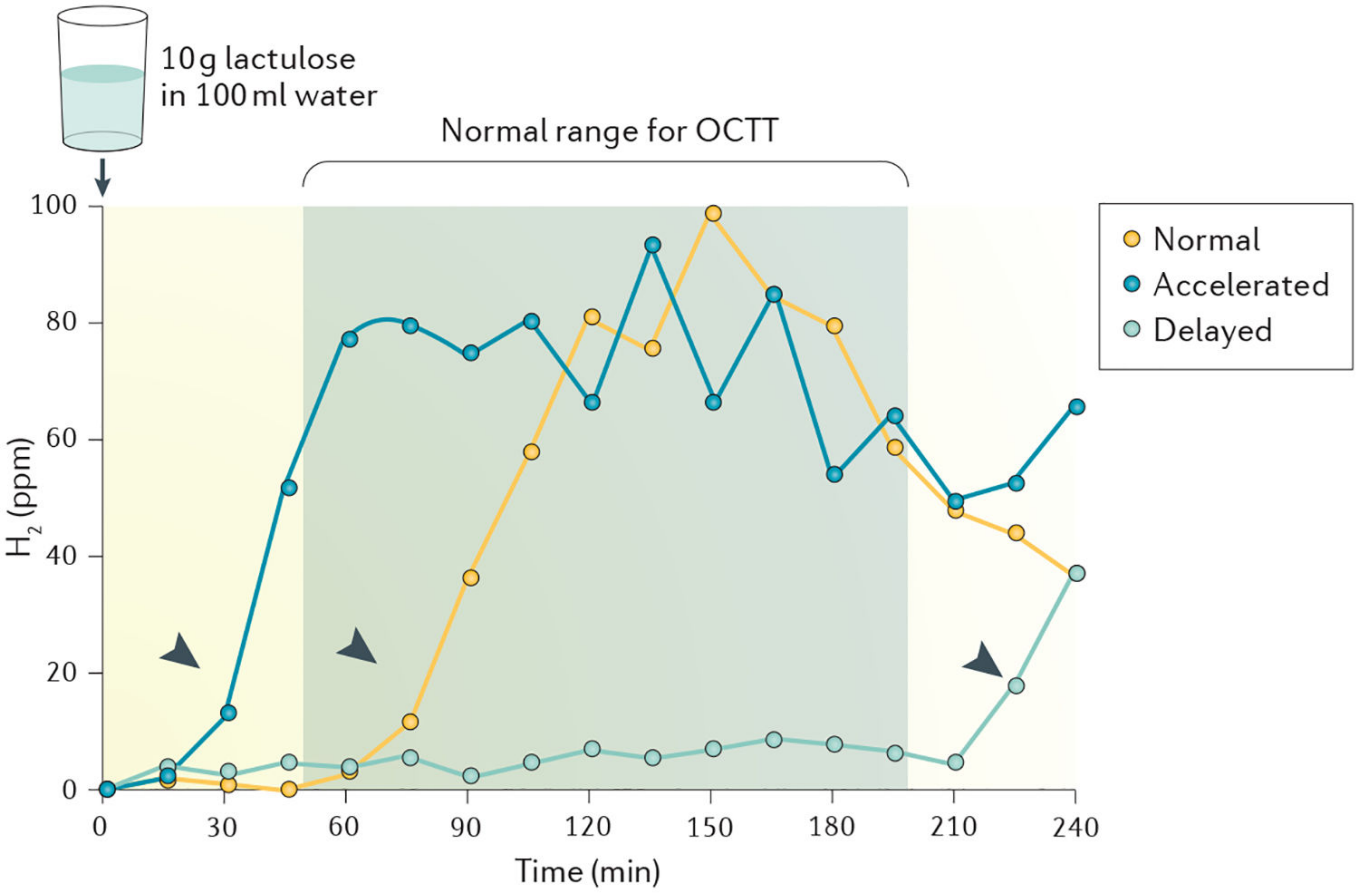
Lactulose H_2_ breath test for measurement of orocaecal transit time. Representative lactulose H_2_ breath tests (LHBTs) are shown for accelerated (30 min), normal (75 min) and delayed (225 min) orocaecal transit times (OCTTs). The test requires H_2_ measurements at regular intervals after ingestion of lactulose. H_2_ values of >10 ppm over basal values followed by at least two subsequent increments (arrows) indicate caecal delivery of the nonabsorbable substrate with subsequent bacterial metabolism. This increase in H_2_ exhalation normally occurs 50–200 min after ingestion of the marker substance (normal range for OCTT marked in grey).

The LHBT is a semi-quantitative test that measures orocaecal transit time using the increase in H_2_ exhalation associated with caecal delivery and subsequent bacterial metabolism of the nonabsorbable saccharide lactulose^56,57^ (FIG. 5). The test can be easily performed and is widely available, inexpensive and not associated with radiation exposure^58^. However, lactulose is not an inert marker; it can accelerate orocaecal transit time through osmotic fluxes into the small intestine^59^, and it also delays gastric emptying time^60^. Moreover, misleading results with falsely short transit times are to be expected in patients with small intestinal bacterial overgrowth, which is particularly problematic because small bowel motility disturbances can cause this condition^61,62^.

Moreover, the LHBT does not specifically measure small bowel transit time; rather, it reflects the summation of gastric and small bowel transit. Gastric emptying of the liquid test solution occurs rapidly and might be negligible in healthy individuals. However, in patients with gastrointestinal motility disturbances, gastric emptying may markedly influence the measured orocaecal transit time. This problem could be overcome by combining the LHBT and the ^13^C-acetate GEBT such that small bowel transit time can be calculated as the difference between orocaecal transit time and the gastric emptying time^63^.

• Small bowel manometry is the reference method for evaluation of intestinal contractile patterns.

Catheter-based manometry with multiple pressure sensors permits detailed assessment of small bowel contraction patterns^3^ (FIG. 4). Manometry sensors are usually placed in only the proximal small bowel (duodenum and proximal jejunum) for practical reasons, and tracings from these segments are assumed to reflect motility of the total small intestine^3^, although this aspect has not been tested rigorously. Ambulatory investigations are performed over 24 h by some centres, while other centres perform stationary manometry with recordings over 3–4 h in the fasting state and for an additional 2 h after ingestion of a test meal^3^. Manometry can reveal low-amplitude contractions or disorganized contractile patterns or normal amplitudes, frequencies and patterns of contractions^3^ (FIG. 4).

## Investigation of colonic motor function

### Indications and clinical importance

• Severe constipation refractory to conventional treatment and not explained by common imaging techniques is the main indication for colonic motor function testing. Certain measurements of colonic motility might provide useful information in a subset of patients with diarrhoea.

Severe colonic dysmotility usually impairs propagation of luminal contents and is consequently associated with slow-transit constipation. In a subset of patients with diarrhoea, relevant alterations of colonic motility can be identified, for example, increased frequency of high amplitude propagated contractions during the day and/or after a meal^3^. Moreover, colonic scintigraphy or radiopaque marker (ROM) transit has been shown to differ between subtypes of functional disorders of the lower gastrointestinal tract and healthy individuals^64,65^. Transit was generally accelerated in diarrhoea and delayed in constipation, confirming that motor dysfunction is of pathophysiological importance. Thus, colonic transit measurement could identify subgroups more likely to respond to treatment directed at dysmotility.

• Evacuation disorders should be excluded as a potential cause of constipation symptoms before intraluminal tests of colonic motility are considered.

A meta-analysis published in 2013 suggested that ~50% of patients with chronic constipation have dyssynergic defecation according to anorectal manometry^66^. In comparison, ~60% of patients with dyssynergic defecation have delayed colonic transit^67^, which can be secondary to the evacuation disorder. Colonic transit could accelerate, and symptoms can improve or even resolve with treatment of the evacuation disorder^68,69^. Thus, delayed colonic transit does not necessarily reflect colonic inertia and does not imply a colonic motility disorder as the sole cause of constipation. Moreover, anatomical alterations such as large rectoceles or mucosal prolapse can impair stool evacuation. Both dyssynergic defecation^70^ and anatomical alterations require specific treatments and should be identified before elaborate investigation of colonic motility.

• Colonic transit tests are required to distinguish normal from slow-transit constipation.

Clinical markers do not predict slow-transit constipation reliably. In particular, stool frequency is a poor surrogate for transit even in those with reduced stool frequency^71,72^. Hard stool (form 1 or 2 on the Bristol Stool Chart) predicts delayed versus normal transit, but only a moderate correlation exists between stool form and whole-gut or colonic transit time in adults with constipation^71^. Moreover, normal-transit constipation has been observed in >70% of patients with constipation-predominant IBS (IBS-C) or functional constipation^64,73^. In ~5% of patients, colonic transit was even accelerated. Vice versa, a subset of patients with IBS with diarrhoea (IBS-D) had delayed colonic transit^64,73^. Transit tests are, therefore, required to identify slow colonic transit and can optimize the choice of treatment^74^.

• Colonic scintigraphy and ROM can provide initial information to differentiate between diffuse and localized colonic dysmotility and/or evacuation disorders. However, transit measurements alone are not diagnostic of evacuation disorders and require confirmation by specialized tests of evacuation.

**Figure 6 |:**
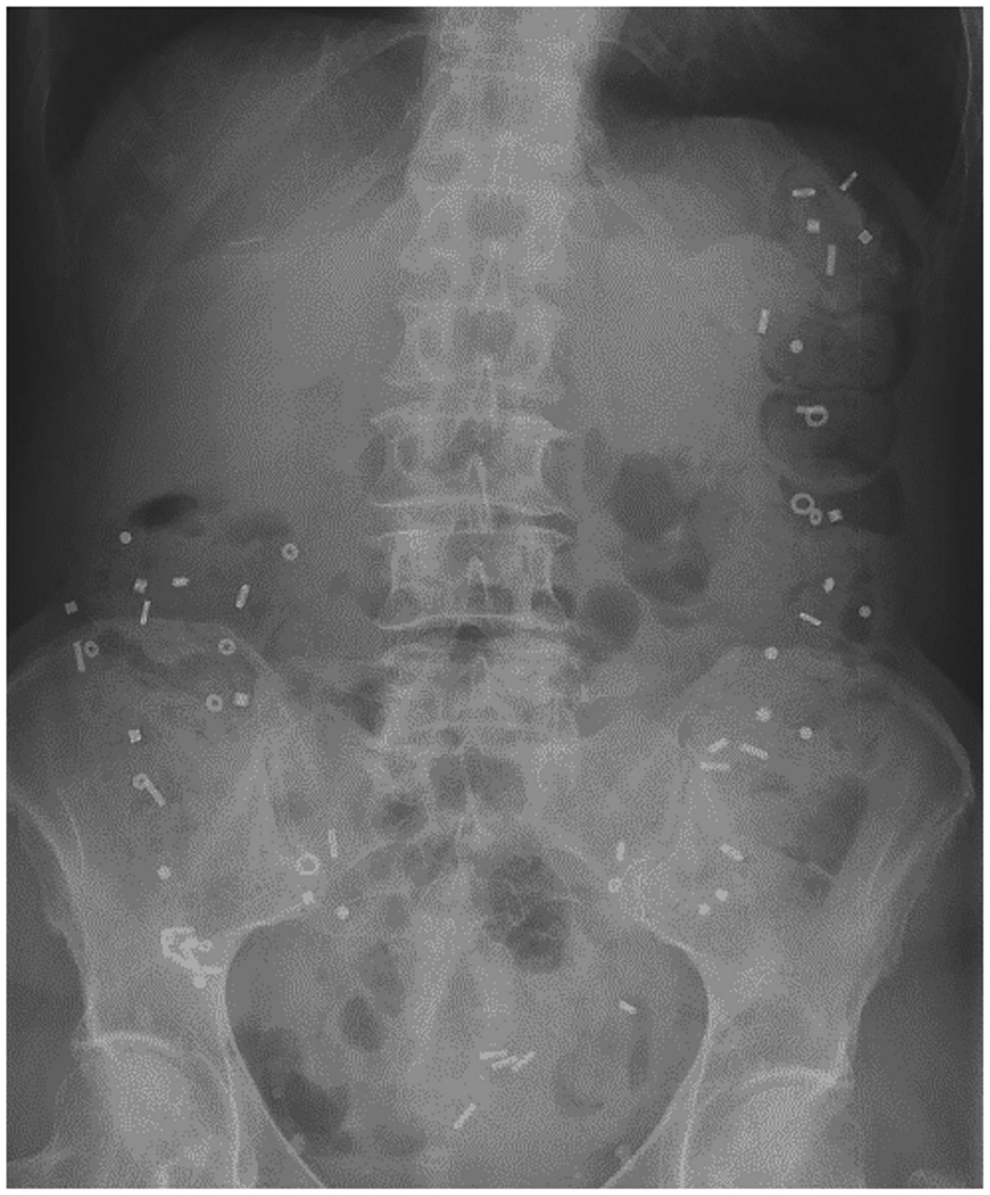
Assessment of colonic transit time with radiopaque markers. A radiopaque marker test of a patient who ingested 10 markers every morning for 6 days is shown. The plain abdominal radiograph was taken on day 7 and shows that all 60 markers are retained; accordingly, colonic transit time is ≥144 h ((number of retained capsules × 24 h)/(number of capsules ingested per day)). Normal values include colonic transit times ≤70 h in a mixed population, ≤50 h in men and ≤70–106 h in women. Note that in this case, the markers are evenly distributed throughout the colon, which is regarded as typical of, but is not completely specific for, slow-transit constipation.

Regional scintigraphic transit profiles and distribution of ROM can give initial information on the pathophysiology of constipation^4,75–77^. Retention of ROM in the entire colon is expected in slow-transit constipation (FIG. 6), whereas concentration of ROM in the rectosigmoid suggests an evacuation disorder. Accordingly, transit tests can help direct treatment: if overall transit is delayed, prokinetic treatment might be indicated; if overall transit is normal, patient education, dietary advice and/or osmotic laxatives usually suffice. If dyssynergic defecation is present, biofeedback training is indicated^70,78^. However, transit can be slow in disorders of rectal evacuation, such that specialized tests such as anorectal manometry, the balloon evacuation test or defaecography are required to confirm functional or structural causes of evacuatory dysfunction^67^. Moreover, even if transit tests suggest that a specific segment of the colon is responsible for delayed transit, in the absence of localized megacolon, experts advise against segmental colonic resection in treatment-refractory slow-transit constipation^79^.

• Invasive therapeutic measures for severe constipation, that is, subtotal colectomy, require proof of colonic dysmotility. In such patients, colonic transit tests are mandatory. Tests of colonic contractility are desirable, including measurement of colonic tone or compliance in some cases.

**Figure 7 |:**
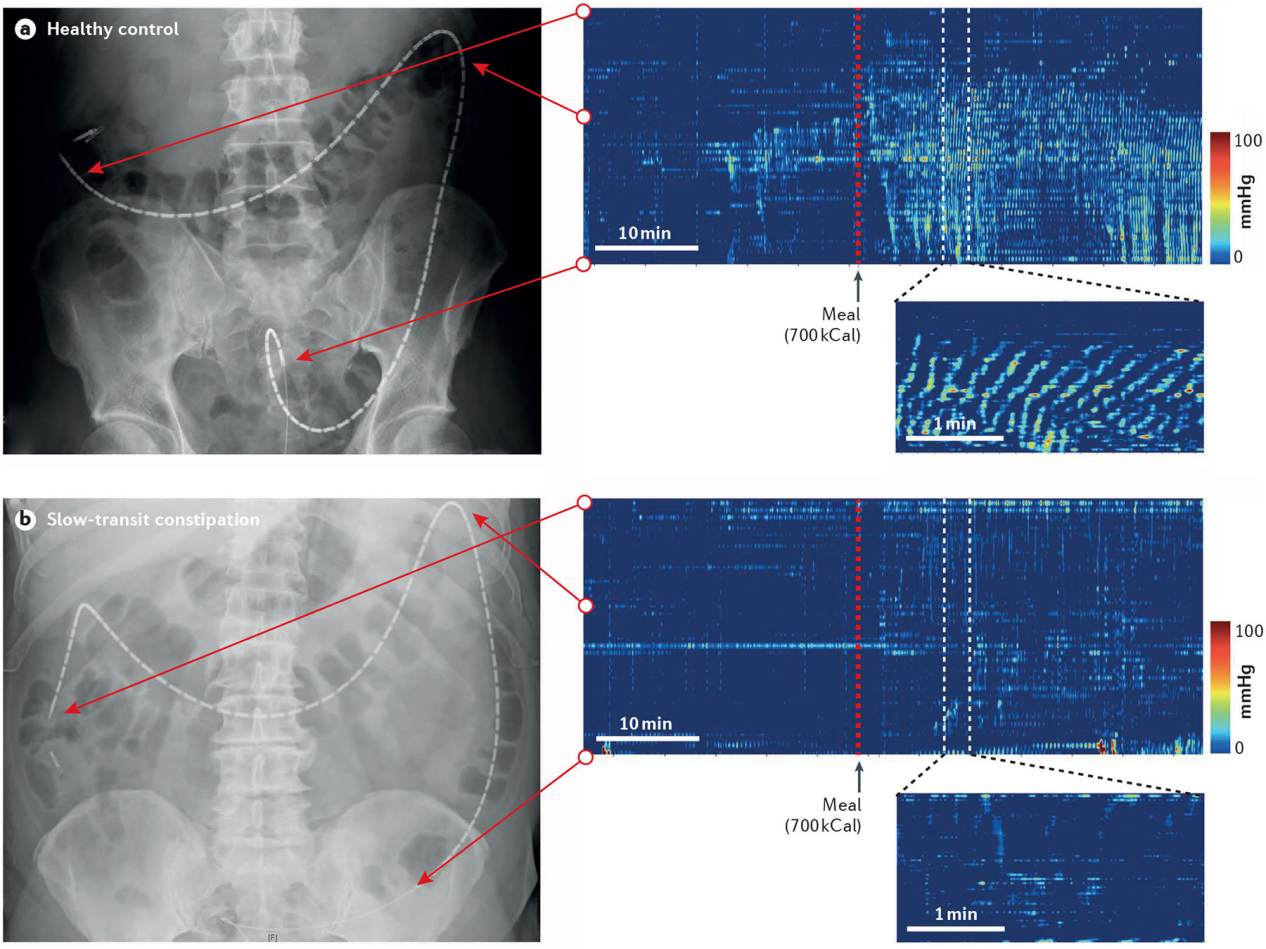
Example colonic high-resolution manometry. Colonic high-resolution manometry recordings in a healthy individual (part **a**) and a patient with slow-transit constipation (part **b**) are shown. Note the physiological increase in colonic contractility that occurs within minutes after the test meal. In the patient with slow-transit constipation, the frequency and amplitudes of colonic contractions are markedly reduced and the motor response to feeding is virtually absent.

**Figure 8 |:**
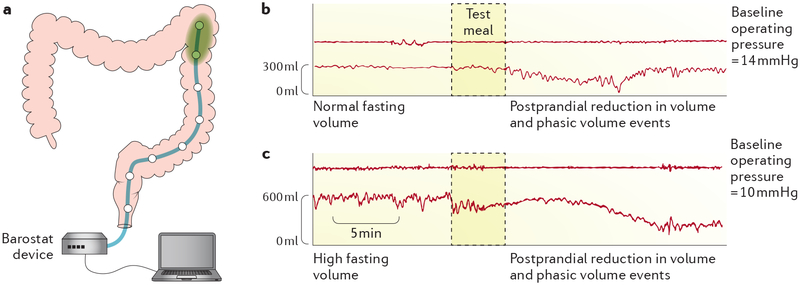
Assessment of colonic tone using a barostat device. The barostat balloon is placed into the colon endoscopically (part **a**). The barostat device keeps intraballoon pressure at a pre-set level chosen to ensure apposition of the balloon to the colonic wall without relevant distension. Phasic and tonic contractions therefore induce a decrease in baseline balloon volume. The panels show phasic and tonic contractile activity measured under constant pressure conditions in the colon of a patient with slow-transit constipation (part **b**) and in the colon of a patient with chronic megacolon (part **c**). Note the large colonic volume (indicating low tone) during fasting in part **c** and the persistence of phasic contractile activity despite the low colonic tone.

International guidelines agree that subtotal colectomy for treatment of chronic constipation is indicated in only patients with severe disease who are refractory to conservative treatment^49,80,81^. Proof of colonic dysmotility is a prerequisite. In patients with slow-transit constipation as documented by transit tests, multiple failed therapeutic trials are used by many centres as an indication for subtotal colectomy^3^. In other centres, a diagnosis of colonic inertia on the basis of colonic contractility testing (FIGS 7,8) is required before subtotal colectomy because some patients with slow-transit constipation have normal colonic contractility, tone and compliance and normal responses to pharmacological stimulation with intraluminal bisacodyl or intra venous neostigmine according to barostat manometry^3^. Major upper gastrointestinal motility disturbances negatively influence the therapeutic outcomes of patients undergoing colectomy^52,82^ and should therefore be excluded. As shown by a small but rigorous study in 18 children, high-resolution colonic manometry might be able to identify underlying neuropathy as suggested by the absence of motor quiescence between bisacodyl-induced high-amplitude propagating contractions; this finding was associated with histologically proven neuropathy (positive predictive value 92%; negative predictive value 100%)^83^. Another study using conventional manometry was unable to classify specific manometric findings as reflective of myopathic or neuropathic abnormalities in patients with colonic motility disorders^84^. Future studies are required to confirm whether high-resolution manometry findings can be used to differentiate aetiologies of colonic motility disorders.

• Measurement of compliance and tone by barostat confirms overt megacolon identified radiologically and can identify less-severe cases of chronic megacolon.

The characteristic feature of chronic megacolon on barostat measurements is an excessively high fasting volume (FIG. 8), which suggests low colonic tone^85^, and a markedly increased colonic compliance. A colonic balloon volume >300 ml at a distension pressure of 20 mmHg was found to be virtually diagnostic of chronic mega colon, such that this measure can be used for diagnostic purposes in patients with clinical suspicion of chronic megacolon or when the imaging studies are equivocal. The same observations are also pertinent in syndromic megacolon and in multiple endocrine neoplasia type 2B syndrome^86^.

### Recommended diagnostic approaches

• ROM studies and colonic scintigraphy are best suited for measurement of colonic transit time.

Scintigraphy can evaluate both regional and overall colonic transit, and depending on the method used, it can be performed as part of a whole-gut transit study over 48 h or 72 h, incorporating assessment of gastric and small bowel transit also^4^. This method provides accurate and quantitative results for colonic transit time but requires highly specialized personnel, is expensive and has limited availability.

ROM studies, on the other hand, can be performed easily and are inexpensive and widely available but are less well standardized across centres, and the availability of quantitative results depends on the technique chosen. Colonic transit time can be quantified after an equilibrium between daily marker output and input has been achieved^87^, which requires ingestion of radiopaque markers and obtaining an abdominal radiograph at specified times. Several validated variations are available. One approach involves ingestion of 20 markers on day 1 and counting the remaining markers on day 5, with >5 remaining markers implying delayed transit^88,89^. In other variations of the ROM test, a fixed number of ROMs are ingested over several days (for example, 24 markers on days 1–3) with abdominal radiography on the following day^75^. Other established protocols use marker ingestion for 4 days, or preferably, 6 days^87^; accordingly, radiography is performed on either day 5 or day 7 (FIG. 6). Patients need to abstain from laxatives for 2 days before and throughout the test. Thus, the long duration of the test hampers compliance, particularly in patients with severe symptoms. Still, decreasing the duration of the testing period is hardly sensible because in mixed populations, mean colonic transit time is 30–40 h with an upper limit of normal of 70 h (REF. 4). In women, a colonic transit time of up to 106 h has been reported to be normal^90^.

• The WMC can be used as an alternative to assess overall (though not regional) colonic transit.

To calculate the colonic transit time, the WMC uses pH pattern and temperature drop or loss of signal to determine ileocaecal passage and evacuation of the capsule, respectively (FIG. 3). Large studies have shown good agreement between the WMC and ROM or scintigraphic studies^54,91,92^. Accordingly, the technique is FDA approved for the evaluation of colonic transit time in patients with chronic idiopathic constipation^93^.

• Colonic manometry (preferably of high resolution) is the reference method for evaluation of colonic contractile patterns.

Colonic motor activity is characterized by phasic or brief contractions and tonic or sustained contractions. Only the former can be assessed adequately by manometry (FIG. 7). Stationary laboratory-based manometric studies conducted for up to 6 h record fasting and post-prandial phasic contractions, as well as colonic tone and compliance, when a barostat assembly is used in addition to manometry (FIG. 8). Ambulatory 24 h studies usually measure only phasic contractions^3^. Conventional manometry has identified isolated pressure waves, propagated low-amplitude and high-amplitude pressure waves (the latter (>75–116 mmHg) being of particular importance for movement of contents across the colon), simultaneous pressure waves (associated with neuropathy in children but not in adults), retrograde pressure waves and periodic colonic and rectal motor activity with bursts of phasic and tonic pressure waves^3,94^. However, it has been shown that high-resolution manometry with closely spaced pressure recording sites <2 cm apart are mandatory to avoid gross misrepresentation of the frequency, morphology and directionality of colonic propagating sequences^95^.

• A barostat enables the assessment of colonic compliance, tone and phasic contractility.

The assessment of colonic compliance and tone requires a barostat device with a balloon placed into the colon endoscopically (FIG. 8). The barostat keeps intraballoon pressure at a pre-set level chosen to ensure apposition of the balloon to the colonic wall without relevant distension. Changes in baseline balloon volume thus reflect changes in colonic tone^3^. Because the barostat can detect phasic contractions that are non-lumen occluding, this technique is also more accurate than manometry for detecting phasic contractions when the colonic diameter is increased (>5.6 cm)^3^ (FIG. 8). Colonic compliance is a measure of the ease with which the colon can be distended and can be evaluated by recording changes of balloon volume in response to stepwise (usually 4 mmHg) increments of intraballoon pressure to 44 mmHg.

The physiological increase in colonic tone in response to a standard meal has been well characterized and varies among the segments of the colon. In the descending colon, a <15% increase in tone after a meal suggests a relevant colonic motility disorder^3^.

## Additional tests

### Tests of neuromuscular function and structures

• The clinical utility of the tests specified in Table 1 is limited or subject to ongoing studies.

Additional tests for assessing gastrointestinal motility have been proposed (TABLE 1) and are the subject of ongoing study or are available at a few centres. For example, gastric mucosal labelling with single-photon emission CT (SPECT) imaging can measure gastric volumes and accommodation. SPECT has been well valid ated^96–98^ and used in thousands of patients in select clinics but is still not widely available. Similar information on gastric motor functions can be obtained by MRI (TABLE 1).

• Limited data on the amplitude of gastrointestinal contractions can be obtained using a WMC.

Apart from pH and temperature, the WMC also records pressure throughout the gastrointestinal tract. However, with a single pressure recording port that traverses the gastrointestinal tract, the WMC cannot identify physiological or pathological motor patterns, which are essential for diagnosing neuropathic gastrointestinal motility disturbances (discussed earlier). Nevertheless, limited data on the amplitude of gastrointestinal contractions can be obtained by a WMC (FIG. 3), and the presence (though not the propagation) of MMC phase III events can be detected with reasonable sensitivity^99^.

• In selected cases with severe disease, full-thickness biopsy could be useful for therapeutic decisions.

Conventional mucosal biopsy samples obtained endoscopically do not contain relevant muscular and neuronal structures, in particular, they lack the muscularis propria and the myenteric plexus. Thus, gastrointestinal neuromuscular disturbances, including those affecting the interstitial cell of Cajal (ICC), can be diagnosed histologically using only full-thickness biopsy samples that are usually obtained surgically. Because of the invasiveness of the procedure, histological investigations are limited to patients with severe disease (unless full-thickness biopsy samples are available from previous surgery). Moreover, clinically relevant information can only be obtained with expert evaluation. The London Classification^100^ classifies gastro intestinal neuromuscular pathology on the basis of defined histopathological criteria derived from previous guidelines and presents indications, recommendations for safe acquisition of tissue, histological techniques and reporting and referral guidelines. Data on the ICC and other enteric system markers from a cohort of patients with gastroparesis and nondiabetic control patients undergoing bariatric surgery can help provide normative values for research and clinical use^101^. Certain histopathological findings, such as ICC loss, were found to correlate with gastric emptying rates in diabetic gastroparesis^102^, and other disorders, such as enteric ganglionitis or myositis, can be the rationale for immunosuppressive treatment^103^. For the stomach, there are only preliminary data suggesting that histological findings can guide treatment^104^. An endoscopic method to obtain myenteric plexus samples for histopathological assessment has been described^105^.

### Other emerging technologies

Although the techniques described earlier are used to measure gastrointestinal transit and contractility or to assess morphological alterations of neuromuscular structures of the gastrointestinal tract, there are other emerging techniques that will probably add valuable information on the classification of gastrointestinal motor disturbances in the future. These techniques concentrate on electrophysiology, release of neurohormonal transmitters from the mucosa, auto immune and inflammatory markers and measurement of autonomic function.

#### High-resolution electrical mapping.

The myoelectric signal of the stomach can be investigated non-invasively using cutaneous electrogastrography (cEGG, TABLE 1)^106,107^. The cEGG profile is disturbed in gastroparesis, probably owing to loss or dysfunction of ICCs. In fact, cEGG has been used clinically for decades and has demonstrated associations between arrhythmias and gastroparesis. However, it is fundamentally limited by its summative nature, low signal quality and incomplete sensitivity and specificity^106–108^. High-resolution electrical mapping has emerged and involves electrodes placed on the stomach at laparoscopy. This technique provides superior spatial data on arrhythmic patterns and mechanisms and has revealed the surprising complexity of gastric arrhythmias^108^. Dysrhythmias include abnormalities of initiation (stable ectopic pacemakers and unstable focal activities) and conduction (retrograde propagation, wavefront collisions, conduction blocks and re-entry) and operate across bradygastric, normal and tachygastric frequencies^108^. Studies in small groups of patients with functional nausea and vomiting or gastroparesis identified slow-wave dysrhythmias in all but one participant^109,110^. Arrhythmias were similar in both patient groups, indicating that they could be spectra of the same disorder^109^. To date, the clinical use of high-resolution mapping is hampered by its invasiveness because it requires general anaesthesia and laparoscopy; however, minimally invasive intraluminal electrical mapping is under development.

#### Biomarkers.

Both functional gastrointestinal diseases and defined gastrointestinal motor disorders such as gastroparesis and CIPO can occur after infections^1,2,111^. Evidence is accumulating that the pathophysiology in these patients is driven by impaired intestinal barrier function, which could cause low-grade mucosal inflammation associated with altered control of or damage to the enteric nervous system^112–116^. These new data are an intriguing and promising field of research. However, there are so far no clinically established mucosal or systemic markers that enable prediction or diagnosis of neuronal dysfunction or loss.

#### Autoimmune mechanisms.

In another subset of patients with gastrointestinal motor disorders, auto-immune mechanisms have been described that lead to impairment of neuromuscular structures and/or function. For example, enteric ganglionitis and subsequent destruction of enteric neurons in paraneoplastic CIPO are frequently associated with anti-Hu antibodies directed against nuclear structures of neuronal cells^117,118^. Antibodies against neuronal voltage-gated calcium and potassium channels, antibodies against the acetylcholine receptor, other neural autoantibodies and other antibody markers of organ-specific autoimmunity (thyroid or gastric parietal cell specificities) have also been described in patients with autoimmune dysmotility^118^.

#### Autonomic dysfunction.

Autonomic dysfunction is another important cause of major gastrointestinal motor disorders. For example, diabetic gastroparesis is largely attributed to autonomic neuropathy, although several other pathophysiological mechanisms, in particular, loss of ICCs, contribute to the impairment of motor function^9,101^. In one study, more patients with autonomic dysfunction appear to have rapid rather than delayed gastric emptying as a potential cause of gastro intestinal symptoms^119^. Heart rate variability measurements have been used successfully for diagnosis of autonomic neuropathy and represent a noninvasive, complementary tool to conventional autonomic testing in the clinic^120,121^. Research has revealed that autonomic dysfunction can also be caused by auto immune mechanisms. Autoimmune autonomic ganglionopathy is a disorder of isolated autonomic failure associated with antibodies to the nicotinic acetylcholine receptor of the autonomic ganglia, which results in severe orthostatic intolerance, syncope, constipation, gastro paresis, urinary retention, dry mouth, dry eyes, blurred vision and anhidrosis^122^. Patients with higher antibody titres have wide spread dysautonomia, whereas those with lower antibody levels can present with, or evolve into, more focal or restricted presentations^122^. Moreover, in patients with autoimmune dysautonomia and gastroparesis, antibodies to glutamic acid decarboxy lase have been described^104^. Importantly, immunomodulatory therapy improved symptoms in a small number of patients positive for antibodies against glutamic acid decarboxy lase who had been refractory to approved drug and device therapies^104^. Thus, some emerging diagnostic techniques could establish new therapeutic options.

## General considerations

• Adherence to standardized and adequately validated study protocols is necessary.

Standardized study protocols validated in a large number of healthy individuals and patients are only available for some motility tests. For instance, WMC testing in clinical practice follows a fixed protocol that involves ingestion of the WMC immediately after consumption of a defined test meal (260 kcal nutrient bar), a 6 h interval before ingestion of the next meal, avoidance of strenuous or vigorous exercise and return of the data receiver after 5 days^93^. For scintigraphic evaluation of transit times and ^13^C-labelled *S. platensis* GEBT, well-validated protocols are also available^31^. For many of the other tests, there are various test protocols, and validation has frequently been performed in a low number of individuals.

• Patient preparation for testing of gastrointestinal motor function usually requires overnight or prolonged fast and avoidance of medications that affect gastrointestinal motility.

Under physiological circumstances, motor patterns of the entire gastrointestinal tract rapidly adapt to food intake. For instance, within minutes after the start of a meal, the proximal stomach accommodates, small bowel motility changes from the cyclic interdigestive to the fed pattern (FIG. 4), and colonic phasic and tonic contractions increase (FIGS 7,8). The extent and duration of motility changes depend on caloric content and composition of a meal. Thus, to enable standardization, it is essential that patients have been fasting for a sufficient length of time. Furthermore, for tests requiring gastric or intestinal catheter placement, the risk of aspiration is reduced by fasting.

To assess an underlying intrinsic motor dis order, avoidance of medications that affect gastrointestinal motility is required, particularly prokinetic agents, opioids, tricyclic antidepressants and laxatives. The duration of withdrawal depends on the half-life of the drug, but usually, 48–72 h are sufficient. Notwithstanding the above guidelines, it might be necessary to perform motility tests despite ongoing medication if essential long-term medication is concerned or if the effect of the drug on gastrointestinal motility is to be determined.

• For gastric emptying testing, fasting blood glucose should be reasonably well controlled.

A blood glucose concentration >288 mg per dl (16 mmol per l) markedly delays gastric emptying in patients with diabetes when compared with euglycaemia^123^. Thus, it is generally recommended that fasting blood glucose should be <275 mg per dl (15 mmol per l) on the study day. Otherwise, delayed gastric emptying owing to neuromuscular disturbances, for example, diabetic autonomic neuropathy, cannot be distinguished from the effects of hyperglycaemia. Some experts recommend lower thresholds (<180 mg per dl (10 mmol per l))^24^.

• Other factors such as use of medications known to influence gastrointestinal motility (for example, prokinetics, opioids, tricyclic antidepressants, laxatives and others), prior surgery (for example, fundoplication, some forms of bariatric surgery or intestinal resections) and drug abuse (for example, of opioids or cannabinoids) should be detailed in the clinical history and considered when interpreting test results.

Although it is not always possible or reasonable to avoid medications known to influence motility (as already discussed), it is mandatory to consider potential confounders when interpreting test results, for example, the abuse of opioids^124–128^ or cannabinoids^129,130^ or prior fundoplication^131^.

• Behavioural conditions such as rumination syndrome or eating disorders should be considered as a cause of symptoms.

Rumination syndrome with apparently effortless regurgitation of gastric contents into the mouth, caused by contractions of the abdominal wall with subsequent re-swallowing or spitting^1^, is a relevant differential diagnosis in patients who report vomiting and regurgitation. Typically, these patients do not respond to conventional therapy. Eating disorders can be misinterpreted as gastro paresis but can also be associated with gastrointestinal motility disorders^132^.

• There is a marked and unclear overlap in symptoms between patients with gastrointestinal dysmotility and patients with functional gastrointestinal disorders, in whom altered motility is thought to be one among several pathophysiological mechanisms.

Substantial overlap exists in symptoms between gastro paresis and functional dyspepsia and between enteric (including colonic) dysmotility and IBS or functional constipation^13,133^. All functional gastro intestinal diseases are associated with some degree of motor disorder in the gastrointestinal tract. Delayed gastric emptying occurs in ~30% and impaired gastric accommodation in up to 40% of patients with functional dyspepsia^134^. Likewise, colonic transit is delayed in patients with IBS-C and accelerated in patients with IBS-D^64^. In patients categorized as ‘severe IBS’, histopathological alterations regarded as diagnostic for severe motility disorders^100^ (such as inflammation and neuronal degeneration in the myenteric plexus) have been observed^135^.

Consequently, there is a continuum ranging from mild disturbances that can be related to both functional disorders and pure motility disorders to severely disturbed gastrointestinal motility, which is usually attributed to defined diseases such as gastro paresis, CIPO or slow-transit constipation. In most cases, differentiation requires additional dimensions, including clinical characteristics, imaging and psychological traits, or the presence of underlying conditions and diseases that are associated with motility disturbances (for example, diabetes mellitus or Parkinson disease).

## Conclusions

Disturbances of gastric and intestinal motor functions are frequent, and the rational use of gastrointestinal investigations is an important tool to establish the diagnosis and to guide treatment in such patients, but more work is needed (BOX 2). To gain clinically relevant and reliable information, adherence to standardized and adequately validated study protocols is necessary. However, standardized study protocols validated in a large number of healthy individuals and patients are available for only some motility tests, and these include ROM and scintigraphic transit measurements. For several other tests, determination of a widely accepted standard is pending.

Complex, invasive investigations of gastrointestinal motility need to be limited to patients with severe disease and will remain available at specialized gastroenterological centres only. By contrast, noninvasive tests such as ^13^C-GEBT and the WMC are increasingly available, such that knowledge of these tests and about gastrointestinal motility testing in general needs to be spread in the medical community.

## Figures and Tables

**Table 1 |:** Complementary tests of gastrointestinal motor function available* or in development

Test	Principle	Performance	Advantages, disadvantages and miscellaneous
MRI^136–145^	Ingestion of (liquid) meal; information on gastric volume, secretion, emptying and contractions can be derived from repetitive scans, information on OCTT and CTT with prolonged measurements possible	Test meal usually labelled with gadolinium; 3D volume scan (‘static’) for evaluation of gastric emptying, fast (‘dynamic’) 2D scan to assess gastric, small bowel and colonic motility OCTT represented by arrival of head of meal in caecum	Advantages: noninvasive, simultaneous information on different physiological aspects (for example, secretion and emptying) Disadvantages: time consuming, expensive Miscellaneous: preliminary data suggest that it can detect colonic high-amplitude contractions
Gastric barostat^146–148^	Computer-controlled pump controls volume or pressure in large non-compliant bag (>700 ml) placed in fundus via nasogastric catheter; measurements of gastric compliance and/or distensibility and sensitivity in response to distension stimulus or meal are possible	Volume change in response to applied pressure or pressure change in response to applied volume (or a meal) is monitored to assess gastric relaxation (accommodation) and contraction; concurrent grading of subjective symptoms and/or sensitivity (gastric hypersensitivity in 40% of patients with dyspepsia) Gastric relaxation documented after meal or nutrient infusion (accommodation impaired in 40% of patients with dyspepsia)	Advantages: best validated method for gastric tone and sensation Disadvantages: invasive, can cause physical and psychological distress Miscellaneous: MRI and, potentially, other imaging modalities provide indirect, noninvasive assessment of gastric volumes
Abdominal ultrasonography^149^	2D: ultrasonography; indirect measurement of gastric emptying by quantifying changes in antral cross-sectional area or diameter 3D: scanning of entire stomach by continuous translational movement along its long axis and transverse sections of entire stomach; computer-assisted 3D-reconstruction	Ingestion of liquid test meal; sonography at regular intervals for prolonged period (for example, at 15 min intervals for 3 h) 2D: 50% emptying time = time when antral area has decreased to half of its maximum 3D: 50% emptying time = time when gastric volume has decreased to 50% of that immediately after meal intake	Advantages: noninvasive Disadvantages: time consuming and not standardized; 2D offers no good representation of meal distribution in stomach
Proximal gastric HRM^150,151^	Pressure drop in the proximal stomach after application of nutrients is used as a measure of gastric accommodation	Transnasal placement of HRM catheter in the (proximal) stomach; registration of pressure for a prolonged period of time	Advantages: generally available owing to dissemination of oesophageal HRM Disadvantages: invasive and has limited normative data and use to date (studies ongoing)
Impedance planimetry for functional lumen imaging^152–154^	Transnasally or transorally positioned probe with 16 serial impedance electrodes enclosed in a high-compliance bag and a solid-state pressure transducer	Probe is positioned (via endoscopy and/or fluoroscopy) so as to straddle the pylorus; the balloon is then inflated while diameter, cross-sectional area and pressure are measured, allowing calculation of distensibility (by dividing cross-sectional area by pressure at a specific balloon volume)	Advantages: direct measurement of pyloric distensibility; can identify phenotypes not otherwise identified; can be combined with endoscopy Disadvantages: invasive and not widely available; limited normative data; uncertain clinical utility
Cutaneous electrogastrography^106,107^	Myoelectric signal at ~3 cpm waveform frequency is normal; signal amplitude (‘power’) increases after meals; loss or damage of interstitial cells of Cajal that generate and propagate slow waves occurs in disease, which is thought to result in arrhythmias and loss of power	Placement of 3 electrodes in a supine or up to 45° reclined position: recognizable waveforms should be visually identifiable in >15 min (fasting) or >30 min (post-meal); in health, 3 cpm rhythm present ≥70% of the time, with increase in power after meals; in tachygastria, >3 cpm present >30% of the time; in bradygastria, <3 cpm present >30% of the time; nonspecific dysrhythmia (absence of a single predominant rhythm), lack of motor response to meal and >20% total power in the tachygastria range are also considered abnormal	Advantages: noninvasive Disadvantages: summative nature of recordings; poor signal-noise ratio; lack of sensitivity and specificity; validity of technique not confirmed by comparison with direct measurements of gastric contractility or emptying and not widely available Miscellaneous: high-resolution electrogastrography mapping from stomach promising
SPECT^96–98^	Imaging of the gastric wall using intravenous ^99m^Tc pertechnetate with noninvasive SPECT and 3D image analysis	^99m^Tc pertechnetate is taken up and excreted by gastric mucosa; images acquired by gamma camera are reconstructed to produce a 3D representation of the entire gastric volume; predominantly used for evaluation of gastric accommodation	Advantages: noninvasive Disadvantages: available at only a few centres Miscellaneous: can be used to assess drug effects
Endoluminal image analysis^155–158^	Computerized analysis of small bowel images obtained by the endoscopic capsule	Ingestion of endoscopic capsule after overnight fast; ingestion of 300 ml liquid meal (1 kcal per ml) 45 min after gastric evacuation A combination of parameters reflecting wall dynamics and movement of content are used to automatically discriminate normal and abnormal intestinal motor function, which provides further discrimination between hypodynamic and hyperdynamic motor disorders	Advantages: noninvasive technique, operator-independent and higher sensitivity than intestinal manometry Disadvantages: restricted to research and requires further validation
Magnetic pill^159,160^	Small magnet is ingested and tracked by external matrix of magnetic field sensors; can detect movements of capsule induced by contractions; change in dominant contraction frequency used to define segmental gastrointestinal transit times	Stationary system: 16 external sensors used (4 × 4) giving a position defined by 5 coordinates (positions *x*, *y* and *z* and angles θ and φ Ambulatory system now trialled, using 4 sensors contained within an extracorporeal portable detector plate Dominant frequency of 3 contractions per min in stomach changes to 10 contractions per min when magnetic pill enters small intestine and drops to 4–5 contractions per min with ileocaecal passage	Advantages: noninvasive Disadvantages: restricted to research and requires further validation and software development

cpm, cycles per minutes; CTT, colonic transit time; HRM, high-resolution manometry; OCTT, orocaecal transit time; SPECT, single-photon emission CT.

* At a few specialist centres.

## Box 1 | Key advances in gastric and intestinal motility disorders

- Symptoms have poor concordance with gastrointestinal dysfunction on clinical investigations of gastrointestinal motility and function, underlining the need for testing to guide treatment
- Scintigraphy is the reference standard for measurement of gastric emptying; ^13^C-gastric emptying breath tests can be used alternatively
- For all gastrointestinal function tests, adherence to adequately validated, standardized study protocols is crucial
- In patients with therapy-resistant constipation under consideration for colectomy, major disorders of upper gastrointestinal motility and evacuation disorders negatively influence therapeutic outcome and should, therefore, be excluded
- The presence of abnormal gastrointestinal function on clinical investigation can direct management and predict responsiveness to medical therapy in several conditions
- Valid reference values are available for many investigations of gastrointestinal motility (particularly, gastric, colonic and anorectal function) based on results from healthy individuals and patient data, thus defining definitively pathological results

## Box 2 | Open research questions

- Studies comparing manometry and other clinical investigation of gastrointestinal motility and function with histological findings are required to better understand the pathophysiological basis of severe gastrointestinal dysfunction and the rationale for their treatment
- Clinical investigations that assess sensory function in patients with functional gastrointestinal disorders require validation
- The interactions of gastric and intestinal function and digestion are poorly defined in health and disease; tests that combine modalities could provide unique insight
- Outcome studies are required to assess indication for new interventions (for example, pyloric botulinum toxin injection, endoscopic pyloromyotomy and pyloroplasty) for less well-established dysfunctions (for example, pylorospasm).
